# A compact phage display human scFv library for selection of antibodies to a wide variety of antigens

**DOI:** 10.1186/1472-6750-9-6

**Published:** 2009-01-29

**Authors:** Potjamas Pansri, Nanthnit Jaruseranee, Kuntalee Rangnoi, Peter Kristensen, Montarop Yamabhai

**Affiliations:** 1 School of Biotechnology, Institute of Agricultural Technology, Suranaree University of Technology, Nakhon Ratchasima 30000, Thailand; 2 Department of Molecular Biology, University of Aarhus, Science Park, DK-8000 Aarhus C, Denmark

## Abstract

**Background:**

Phage display technology is a powerful new tool for making antibodies outside the immune system, thus avoiding the use of experimental animals. In the early days, it was postulated that this technique would eventually replace hybridoma technology and animal immunisations. However, since this technology emerged more than 20 years ago, there have only been a handful reports on the construction and application of phage display antibody libraries world-wide.

**Results:**

Here we report the simplest and highly efficient method for the construction of a highly useful human single chain variable fragment (scFv) library. The least number of oligonucleotide primers, electroporations and ligation reactions were used to generate a library of 1.5 × 10^8 ^individual clones, without generation of sub-libraries. All possible combinations of heavy and light chains, among all immunoglobulin isotypes, were included by using a mixture of primers and overlapping extension PCR. The key difference from other similar libraries was the highest diversity of variable gene repertoires, which was derived from 140 non-immunized human donors. A wide variety of antigens were successfully used to affinity select specific binders. These included pure recombinant proteins, a hapten and complex antigens such as viral coat proteins, crude snake venom and cancer cell surface antigens. In particular, we were able to use standard bio-panning method to isolate antibody that can bind to soluble Aflatoxin B1, when using BSA-conjugated toxin as a target, as demonstrated by inhibition ELISA.

**Conclusion:**

These results suggested that by using an optimized protocol and very high repertoire diversity, a compact and efficient phage antibody library can be generated. This advanced method could be adopted by any molecular biology laboratory to generate both naïve or immunized libraries for particular targets as well as for high-throughput applications.

## Background

Monoclonal antibodies have become important tools in several fields, including molecular biology, pharmaceutical and medical research, as well as in the treatment of diseases such as cancer and infectious diseases [1-3]. Since the advent of antibody technology, antibody production has moved from hybridoma technology to recombinant DNA methodology. The advantages of recombinant antibodies are several folds, (i) antibodies can be produced in bacteria, yeast or plant [4-6], (ii) immunization is not required and (iii) intrinsic properties such as immunogenicity, affinity, specificity and stability of antibodies can be improved by various mutagenesis technologies [7-9]. In the past two decade, advances in phage display and antibody engineering have led to the development of phage-displayed antibody technology [10,11]. This technology allows one to isolate antibodies directly from diverse repertoires of antibody genes, generating high-affinity binding sites without the constraint imposed by classical method for generating either polyclonal or monoclonal antibody [12-16]. Since the method does not depend on an animal's immune system, antibodies to a wide variety of antigens, including the molecules that cannot stimulate immune system of the animals such as nonimmunogenic, "self", cell surface or toxic antigens, can be generated [16-18]. The antibodies can also be engineered to contain in-built features that suit various downstream applications [19] or converted into functional whole immunoglobulin [20,21]. The antibody genes are expressed and the gene products displayed on the surface of filamentous bacteriophage as fusion proteins [7,11,22-25]. This collection of phages is called a phage display antibody library, where each phage particle displays a single antibody. In order to construct a library, antibody genes are fused to phage genes, thus creating a link between antibody phenotype and its encoded genotype. Antibody genes can be isolated from B-lymphocytes of non-immunized donors, rendering a naïve library which is a valuable source of human monoclonal antibodies against various antigens [26]. Various formats of antigen-binding fragments, including Fab and scFv have been cloned and displayed on phage [27,28]. The advantage of smaller antibody fragments is that they have high tissue penetrability, while maintaining their affinity and specificity [29-31]. They are also easier and faster to produce in recombinant form. However, successful construction of a human antibody phage library has been achieved only by a small number of research groups [10,29,32]. One reason may be because of the complexity and cost of generation of the library, even though there have been some reports describing optimized protocols for the generation of efficient libraries [32,33].

Here we report a simple and highly efficient method for the construction of a compact and highly useful scFv human library. The library was based on the naïve human re-arranged V-genes and assembled through the use of a gene repertoire derived from 140 non-immunized donors. All possible combinations of heavy and light chains, among all immunoglobulin isotypes, were included by using a mixture of primers and overlapping extension PCR. The resulting variable gene repertoire were cloned to form a moderate size library composed of 1.5 × 10^8 ^individual clones from one ligation reaction. This repertoire was used for selection of specific binders to different proteins, a hapten, and complex antigens i.e., viral coat proteins, crude snake venom and cancer cell surface. Binding specificity and sequence diversity among binders were demonstrated.

## Results

### Construction of pMod1 phagemid

**Figure 1:**
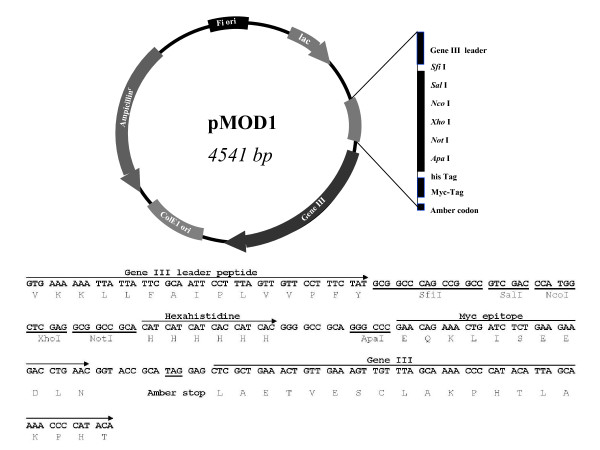
**Map of phagemid vector, pMod1, for the construction of scFv phage-display library**. This 4541-base-pair (bp) vector was modified from pHage 3.2 (Maxim Biotech, Inc.), which was derived from M13. Transcription is under control of the *lac *promoter. The ampicillin resistant gene is for the selection and maintenance of the phagemid. The phagemid carries Fi and ColEi origin of replication for amplification in phage and *E. coli*. The secretion of the scFv fragment is directed by gene III signal peptide. The scFv genes are cloned between *Sfi*I and *Not*I for display on the phage coat protein, pIII. An amber stop codon in frame with and immediately following hexahistidine-tag and Myc-tag permit the expression of scFv as a fusion with gene III in an amber suppressor host (TG1) or as a fusion with only the 6 × His-tag and Myc-tag in a non-suppressor host strain (HB2151). The scFv fragment is fused with hexahistidine tag and Myc tag for affinity purification and immuno-detection, respectively. The coding region of a gene III leader peptide and the multiple cloning sites is shown with the position of 6 × His, Myc tags and amber stop codon. The map was not drawn to scale. The sequences around the cloning sites were shown at the bottom of the figure.

A novel phagemid vector, designated pMod1, for the construction of phage-displayed scFv library was created (Figure 1). This vector was based on the phagemid vector, pHage 3.2 (Maxim Biotech Inc, USA). A multiple cloning site was introduced, containing five restriction recognition sties, of which *Sfi*I and *Not*I were used for the insertion of scFv gene repertoires. The gene III leader peptide was used to direct the secretion of scFv, whereas ampicillin resistant gene was used for the selection and maintenance of the phagemid. The scFv gene was linked to the hexahistidine tag followed by Myc epitope. The hexahistidine tag can be used for one-step affinity purification with immobilized metal affinity chromatography (IMAC), whereas the Myc epitope can be recognized by 9E10 monoclonal antibody for detection. An amber stop codon was introduced between the Myc-tag and gene III, thus allowing production of non-fused scFv by introduction of the phagemid DNA in a non-suppressor *E. coli *strains (HB2151).

### Library construction

**Figure 2:**
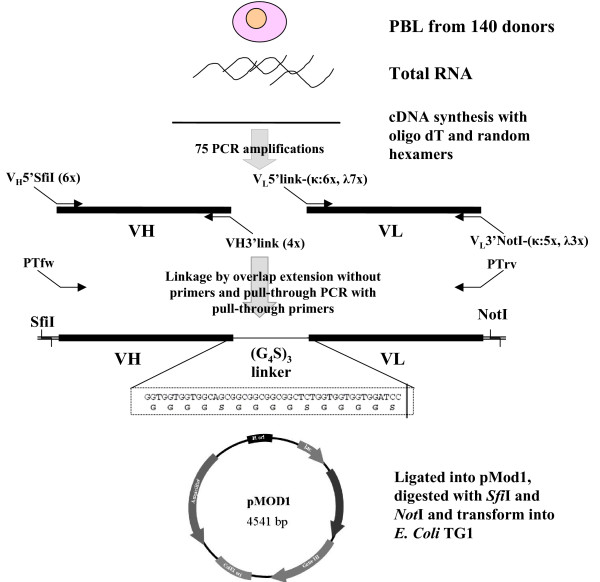
**Schematic outline of the strategy used for the construction of compact scFv library**. The total RNA was extracted from peripheral blood lymphocyte (PBL) of 140 non-immunized healthy donors. cDNA was synthesized by using a mix of random hexamer and oligo dT_18_. The locations of all PCR primers on the two variable region genes are shown. The list of all primers used for the construction of the library is given in Table 1. Three step PCR reactions were performed. The first PCR step comprises 75 reactions for amplification of V gene repertoire. The second and third PCRs link and amplify full-length scFv gene repertoire, which were cloned into pMod1 phagemid. The DNA sequence encoding the flexible linker is depicted in detail.

A diagram that outlines the construction of the compact scFv antibody library is shown in Figure 2. Peripheral blood from one hundred and forty healthy non-immunized donors was collected into four pools, according to different blood groups. These include adults at the age between 17–50, both male and female, in Nakhon Ratchasima province. The bloods were tested and discharged from the Thai Red Cross Society blood donation unit. Total RNA was prepared from the B lymphocytes and pooled together before being used as templates for the construction of V-genes repertoire. A mix of oligo-dT_18 _and random hexamers were used to synthesize cDNA, so that all five antibody isotypes could potentially be represented. In order to reduce amplification bias, we performed 75 independent PCR reactions to amplify V gene segments, using all possible combinations within a primer set (Table 1). The primer sequences, which in theory encompass the entire repertoire of human antibody genes, were obtained from V BASE [34], and modified according to previously published protocols [14,32]. The PCR reactions included six V_H _forward primers (V_H_5'SfiI) paired with four V_H _reverse primers (V_H_3'link) which generated a total of twenty-four reactions; whereas six V_κ _forward primers (V_L_5'link-κ) paired with five V_κ _reverse primers (V_L_3'NotI-κ) generated a total of thirty reactions; and seven V_λ _forward primers (V_L_5'link-λ) paired with three V_λ _reverse primers (V_L_3'NotI-λ) generated a total of twenty-one reactions. The PCRs led to the representation in the repertoire of variable regions derived from all conceivable framework assemblies.

**Table 1:** Primers for the Construction of Human scFv Phage Display Library

**Primer**	**Sequence**
V_H_5'Sfi	5' CCTTTCTATGC*GGCCCAGCCGGCC***ATGGCCCAGGTGCAGCTGGTGCAGTCTGG **3' 5' CCTTTCTATGC*GGCCCAGCCGGCC***ATGGCCGAGGTACAGCTGCAGCAGTCAGG **3' 5' CCTTTCTATGC*GGCCCAGCCGGCC***ATGGCCCAGGTCAACTTAAGGGAGTCTGG **3' 5' *GCCCAGCCGGCC***ATGGCCGAGGTGCAGCTGGTGGAGTCTGG **3' 5' *GCCCAGCCGGCC***ATGGCCCAGGTGCAGCTGCAGGAGTCGGG **3' 5' *GCCCAGCCGGCC***ATGGCCGAGGTGCAGCTGTTGCAGTCTGC **3'
	
V_H_3'link	5' *ACCAGAGCCGCCGCCGCCGCTACCACCACCACC***TGAGGAGACGGTGACCAGGGTGCC **3' 5' *ACCAGAGCCGCCGCCGCCGCTACCACCACCACC***TGAGGAGACGGTGACCGTGGTCCC **3' 5' *ACCAGAGCCGCCGCCGCCGCTACCACCACCACC***TGAAGAGACGGTGACCATTGTCCC **3' 5' *ACCAGAGCCGCCGCCGCCGCTACCACCACCACC***TGAGGAGACGGTGACCAGGGTTCC **3'
	
V_L_5'link-κ	5' *AGCGGCGGCGGCGGCTCTGGTGGTGGTGGATCC***GACATCCAGATGACCCAGTCTCC **3' 5' *AGCGGCGGCGGCGGCTCTGGTGGTGGTGGATCC***GAAATTGTGCTGACTCAGTCTCC **3' 5' *AGCGGCGGCGGCGGCTCTGGTGGTGGTGGATCC***GATGTTGTGATGACTCAGTCTCC **3' 5' *AGCGGCGGCGGCGGCTCTGGTGGTGGTGGATCC***GAAATTGTGTTGACGCAGTCTCC **3' 5' *AGCGGCGGCGGCGGCTCTGGTGGTGGTGGATCC***GACATCGTGATGACCCAGTCTCC **3' 5' *AGCGGCGGCGGCGGCTCTGGTGGTGGTGGATCC***GAAACGACACTCACGCAGTCTCC **3'
	
V_L_5'link-λ	5' *AGCGGCGGCGGCGGCTCTGGTGGTGGTGGATCC***AATTTTATGCTGACTCAGCCCCA **3' 5' *AGCGGCGGCGGCGGCTCTGGTGGTGGTGGATCC***CAGTCTGTGTTGACGCAGCCGCC **3' 5' *AGCGGCGGCGGCGGCTCTGGTGGTGGTGGATCC***CAGTCTGCCCTGACTCAGCCTGC **3' 5' *AGCGGCGGCGGCGGCTCTGGTGGTGGTGGATCC***TCCTATGTGCTGACTCAGCCACC **3' 5' *AGCGGCGGCGGCGGCTCTGGTGGTGGTGGATCC***TCTTCTGAGCTGACTCAGGACCC **3' 5' *AGCGGCGGCGGCGGCTCTGGTGGTGGTGGATCC***CACGTTATACTGACTCAACCGCC **3' 5' *AGCGGCGGCGGCGGCTCTGGTGGTGGTGGATCC***CAGGCTGTGCTCACTCAGCCGTC **3'
	
V_L_3'NotI-κ	5' CAGTCATTCTCGACTT*GCGGCCGC***ACGTTTGATTTCCAGCTTGGTCCC **3' 5' CAGTCATTCTCGACTT*GCGGCCGC***ACGTTTAATCTCCAGTCGTGTCCC **3' 5' CAGTCATTCTCGACTT*GCGGCCGC***ACGTTTGATCTCCAGCTTGGTCCC **3' 5' CTCGACTT*GCGGCCGC***ACGTTTGATATCCACTTTGGTCCC **3' 5' CTCGACTT*GCGGCCGC***ACGTTTGATCTCCACCTTGGTCCC **3'
	
V_L_3'NotI-λ	5' CAGTCATTCTCGACTT*GCGGCCGC***ACCTAAAACGGTGAGCTGGGTCCC **3' 5' CTCGACTT*GCGGCCGC***ACCTAGGACGGTGACCTTGGTCCC **3' 5' CTCGACTT*GCGGCCGC***ACCTAGGACGGTCAGCTTGGTCCC **3'
	
PTfw	5' CCTTTCTATGC*GGCCCAGCCGGCC*ATGGCC 3'
	
PTrv	5' CAGTCATTCTCGACTT*GCGGCCGC*ACG 3'

*Bold fonts indicate sequence complementary to the V-gene segments. Recognition sites for restriction enzymes (*Sfi*I/*Not*I), and linker sequence are italicised.

A total of 33 primers were used for the construction of the scFv library. All primers contained either *Sfi*I or *Not*I recognition sites or linker sequence as illustrated in Figure 2 and detailed in Table 1. The final pull-through PCR could be done with two primers (PTfw & PTrv) compatible to the 5' *Sfi*I or 3'*Not*I segments of the heavy and light chain gene repertoires. We found that it was not necessary to add extra nucleotides 5' to the recognition sites as previous published [32,33], provided that sufficient incubation period (overnight) was performed for each digestion reaction. After the final scFv gene repertoires had been sequentially digested with *Sfi*I and *Not*I, they could be ligated directly into pre-digested and dephosphorylated phagemid. From one ligation reaction and two electroporations, we were able to obtain the final compact scFv library consisting of 1.5 × 10^8 ^different scFv molecules with 0.04% of clones from no-insert ligation.

### Diversity of antibody fragments

**Figure 3:**
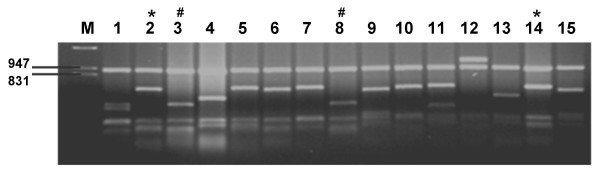
**DNA fingerprinting of random clones from unselected library**. Fifteen scFv gene fragments were amplified by PCR and digested with *Bst*N1 at 60°C for 3 hours. The restriction patterns were analyzed on 2% agarose (* and # indicate similar patterns).

To analyze the diversity of the scFv repertoire and the quality of the primary library, DNA segments encoding the scFv genes from fifteen randomly picked clones were examined. The phagemid DNA of these clones were amplified by PCR and digested with *Bst*NI, and their fingerprint patterns were compared. Thirteen different patterns were identified as shown in Figure 3. DNA sequence analysis of ten different clones revealed that the scFv fragments were all in-frame [see Additional file 1]. The fingerprints of clone3 and clone8 were apparently identical; although their nucleotide and amino acid sequences were different. The variable regions were derived from thirteen different V gene families, including all six V_H _gene families (V_H_1, V_H_2, V_H_3, V_H_4, V_H_5, and V_H_6) and seven V_L _gene families of both kappa and lambda light chain (V_κ_2, V_κ_3, V_κ_4 and V_λ_2, V_λ_3, V_λ_5, V_λ_6). The V-gene segments such as V_H_3DP47, V_λ_DPL16, and V_κ_3DPK22 which are most often used in the natural B cell repertoire [17,33,35] were included in the unselected library as well as other less frequent segments. The CDR3 of V_H _sequences were highly diverse, with lengths between 5 to 19 amino acids. The V_L _sequences had between 8 and 13 amino acids in their CDR3 regions. Thus, the scFv gene fragments were distributed across the full repertoire of antibody germ line genes. The number of different amino acids from germ line varied from 0–24 amino acids, suggesting that both germ line B genes and antibodies from secondary immune responses were included in the library [see Additional file 1]. Analysis of germ line family was done by using Ig BLAST [36], and DNA Plot program [34].

### Selection of scFv antibodies against various antigens

Because the library is compact and a protease sensitive helper phage, KM13 [37] was used during bio-panning, only one or two round of selection were performed on eight different antigens. These antigens include pure proteins [BSA, GST, Amylase], hapten [BSA-conjugated Aflatoxin B1], and very complex antigens [snake venom (cobra and green viper), rabies viruses, and cholangiocarcinoma (bile duct cancer) cells]. To reduce the amount of antigens needed for each bio-panning, all antigens were immobilized on wells of 96-well ELSIA plate (except for cancer cells that were grown on 5-ml flask). The numbers of clones obtained from the first rounds of selection were varied, as shown in Table 2. After the first round of selection with a particular antigen, up to 96 phage clones were picked and their binding specificity determined by phage ELISA. We were able to obtain at least one specific phage clone for all antigens, except for crude green pit viper snake venom. An additional round of panning increased the ratio of binders but did not increase the chance to obtain clones not already detected after the round one. As demonstrated in the case of snake venom, the nine clones that were isolated from the second round of panning were all the same and identical to the clone obtained from the first round of selection. For attenuated virus, six out of seven clones from the second round of panning were identical. The number of clones obtained from the first round of panning varied, depending on the type of the antigens and the number of washings. We found that the most appropriate number of clones after the first round of selection should vary between 100–1,000 colonies. This result suggested that, for a compact naïve library like in this report, one round of selection is sufficient and most appropriate to obtain scFv antibodies with maximal diversity.

**Table 2:** Results of affinity selections with different targets

**Antigens**	**Round of panning**	**Number of clone after 1^st ^round**	**Number of binding phage after 1^st ^round**	**Number of binding phage after 2^nd ^round**	**Number of soluble scFv fragments**	**Number of different scFv producing clone**
Aflatoxin	1	2.5 × 10^2^	4/56	-	3/4	3/3
Attenuated Rabies viruses	2	1.2 × 10^3^	1/96	7/96	8/8	2/8
Amylase	1	8.8 × 10	3/88	-	3/3	NA
BSA	1	2.0 × 10^3^	6/96	-	NA	NA
Cobra snake venom	2	1.4 × 10^4^	1/96	9/96	3/3	1/10
Cholangiocarcinoma cell KKU-100	1	2.2 × 10^3^	2/96	-	NA	2/2
Green pit viper venom	2	5.1 × 10^4^	0/96	0/96	NA	NA
GST	1	4.3 × 10^2^	6/28	-	NA	NA

* The number of positive clones/the number of screened clones.

### Characterization of selected scFv antibodies

**Figure 4:**
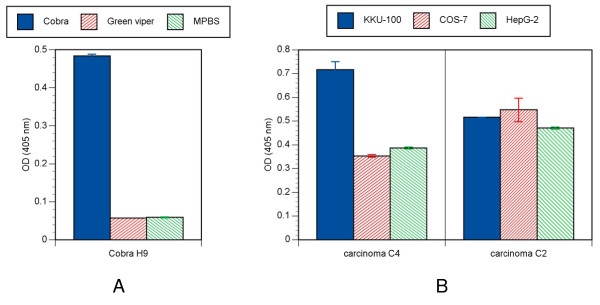
**Binding specificity of selected phage**. Panel **A **shows the specific binding of phage selected from bio-panning against crude cobra snake venom. One hundred microgram of lyophilized crude cobra (*Naja kaouthia*) and green pit viper (*Trimeresurus albolabris*) snake venom were immobilized onto well of ELISA plate and incubated with the representative phage clone. Bound phage particles were detected by ELISA. The average OD_405nm _values and standard errors are shown. Panel **B **illustrates binding activity of two phage clones selected from bio-panning with cholangioma against different cell lines. KKU-100 is a human cholangiocarcinoma (bile duct cancer) cell line; COS-7 is an African Green Monkey SV40-transfected kidney fibroblast cell line; and HepG2 is a human hepatocellular liver carcinoma cell line. The cells were fixed with 4% paraformaldehyde before incubating with individual phage.

The binding of scFv to antigens was highly specific even though the antigens were very complex. For example, the selected anti-cobra venom scFv did not cross react with green viper snake venom (Figure 4, panel A). Another example of scFv specificity is illustrated by the selection against bile duct cancer cells. One of the two clones demonstrated higher specificity for a cholangiocarcinoma cell line, when compared to COS-7 or HepG-2 cells (Figure 4, panel B). This experiment was done without pre-incubation or subtraction with other cell lines, demonstrating that it is possible to obtain highly specific antibodies against a complex cell surface antigen by one round of bio-panning using this compact library.

To determine the binding specificity of selected soluble scFv fragments, *E. coli *HB2151 was infected with specific phage selected on different antigens. HB2151, unlike TG-1, is not able to suppress the amber stop codon upstream of the gene III sequence in pMod1 vector, thus resulting in expression of non-fused scFv. Most of these clones produced functional antibody fragments which were specific to their antigen, readily detected in cell culture supernatants by ELISA, as summarized in Table 2.

DNA sequence analysis of selected scFv clones (Table 3) revealed that V_H _genes were derived from three of the six V_H _gene families (nos. 1, 3, and 4). Families 1 and 4 have been shown to be predominating in previous reports [33,35]. Comparison of the six V_H _sequences with their closest germline V sequence using IgBLAST [36] and V BASE [34] revealed that they were from 6 different germ-line genes with various numbers of amino acid differing compared to the germ line sequences, ranging from 1–14. The V_L _genes were from four V_λ _gene families (nos. 1, 2, 3, and 6) and four different germ-line genes. It is interesting that only one V_κ _gene (V_κ_1) family was obtained and they were both from the same germ line genes, but different in the number of different amino acids. The V_κ _1 family has previously found to be most frequently used in other reports as well [33,35]. The preferential usage of V_H _gene family 1 and 3, especially segment DP47 has previously been observed [17,33], this segment is most often used in nature [17].

**Table 3:** Sequence diversity of antibody fragments selected against four different antigens

**Clone**	**Family**	**CDR3**	**Germline**	**Amino acids differences from germline**
**V_H _gene**				
Aflatoxin C3	VH1	ADDYGSGSYGFDY	IGHV1-3*01 (DP25)	1
Aflatoxin C5	VH3	SRVGLWGPRYYYYYGMDVW	IGHV3-23*04 (DP47)	4
Aflatoxin D2	VH1	GGPLDY	IGHV1-45*02 (DP4)	5
Rabies D7	VH1	GGNFDY	IGHV1-18*01 (DP14)	2
Rabies B5	VH3	GYATFDY	IGHV3-23*01 (DP47)	7
Cobra D11	VH4	HGRDTSGYTMDYFDS	IGHV4-59*07 (H4)	14
Carcinoma C4	VH3	DRGKYPGDGMGV	IGHV3-23*01 (DP47)	6
**V_L _gene**				
Aflatoxin C3	VK1	QQSYSTPYA	IGKV1D-39*01 (DPK9)	4
Aflatoxin C5	VL3	QVWDRDSRTIV	IGLV3-9*01 (V2-6)	16
Aflatoxin D2	VL2	SSYAGSNNLV	IGLV2-8*01 (V1-2)	3
Rabies D7	VL1	AAWDDSLSGPV	IGLV1-47*01 (DPL3)	2
Rabies B5	VK1	QQYSYNPYT	IGKV1D-39*01 (DPK9)	1
Cobra D11	VL6	QSYDSSNRV	IGLV6-57*01 (V1-22)	5
Carcinoma C4	VL1	AAWDDSLNGYV	IGLV1-44*01 (DPL2)	2

* CDR3 sequence and V-gene segment usage for both heavy and light chains of the selected antibody are reported. Sequence analysis was done by Ig BLAST [36] and DNA Plot program [34] (shown in parenthesis).

Analysis of the CDR3 regions of both V_H _and V_L _shows considerable variability in their length, ranging from 6–19 amino acids, which is similar to the distribution of the length of CDR3 in unselected library. The variations of the CDR3 length in V_H _genes (6–19) were higher than that of the V_L _genes (9–11). The V_H _CDR3 length and V_H_-gene segment frequency is similar to those observed in natural antibodies [38].

We have also observed one phenomenon similar to chain promiscuity, where one light chain was found in combination with different heavy chain. The rabies B5 clone and the Aflatoxin C3 clone used the same germ-line V_L _gene (V_κ_1, IGKV1D-39*01), but different V_H _genes (Table 3). However, the CDR 3 regions of the two V_κ _genes were different. This result suggested the framework of this V_κ _gene segment is favourable for phage-display format. The number of different amino acids from germ line of the six selected clones varied from 1–16 amino acids, as seen in unselected library. This result suggested that both germ line B genes and antibodies from secondary immune responses were selected from the bio-panning. The summary of DNA sequence analysis of all six selected scFv clones is shown in Table 3.

### Analysis of Aflatoxin-scFv antibodies by competitive ELISA

**Figure 5:**
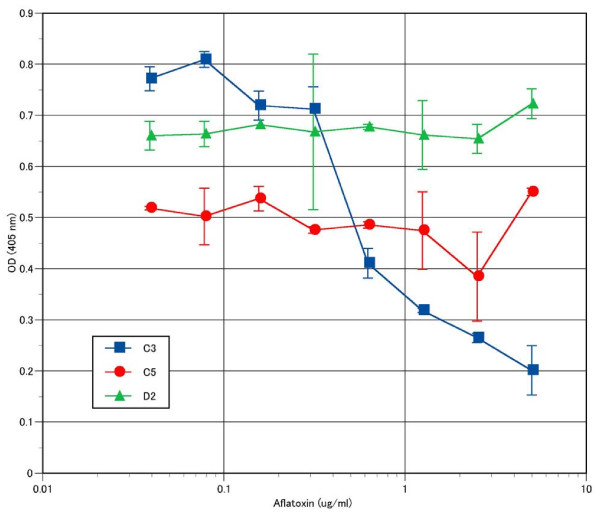
**Inhibition ELISA of anti-Aflatoxin B1 antibody**. Various 1:2 dilutions of soluble Aflatoxin B1 from 5.0–0.039 μg/ml in 2% MPBS were incubated with three selected Phage (C3, C5, D2) at 37°C for 30 min before adding into wells of Immuno 96 MicroWell™ Plates, coated with 4 μg/ml BSA-conjugated AlatoxinB1. After 1 hr of incubation, the plates were washed three times with PBS-T followed by 3 times with PBS. Bound phage were detected with anti-M13 phage-horseradish peroxidase (HRP) conjugate, using ABTS (2,2-azino-di-3-ethyl-benzthiazoine-6-sulfonate) as a substrate. The average absorbance at a wavelength of 405 nm and S.D. are shown.

Normally antibodies are selected by bio-panning against hapten conjugated to immobilizing molecules such as BSA, thus in many cases anti-hapten antibodies cannot recognize free haptens [39]. However, in case of haptens such as Aflatoxin, it is essential for most applications that the antibody recognizes the soluble form of the hapten [40,41]. In this report we demonstrated that by using our compact scFv library and a simple bio-panning method, we were able to obtain three phage clones that could interact specifically with BSA-conjugated Aflatoxin B1, and one of these clones could interact specifically with soluble form of AflatoxinB1, as demonstrated by inhibition ELISA (Figure 5). This type of assay has been used as an indirect method to estimate the binding affinity of an antibody [42].

## Discussion

Phage display antibody technology has become increasing popular for creating binding sites for use in all areas of research, and in medical and industrial applications. There are several examples of successful isolation of antibodies against various antigens from different phage displayed antibody libraries [11,17,33,35,43], including high throughput selection [44]. Unfortunately, the libraries are not available commercially; any laboratories that are interested in using this technique are required to construct a library by themselves, or obtained existing libraries for research with restrictions. Of the two types of phage antibody libraries (immunized and non-immunized libraries), the nonimmune library is of more general use, because it can be applied to generate antibodies to any desired antigen [12,28,33,35]. These "naive" libraries normally has been constructed from the light-chain and heavy-chain IgM-V-gene pools of B cells isolated from peripheral blood lymphocytes, bone marrow, or spleen cells of nonimmunized healthy donors [13,17,44]. However, it has been reported that the diversity of the library could also be maximized by using random hexamers to prime cDNA synthesis, so that all five antibody classes could potentially be represented [35]. Since the whole antibodies cannot be functionally expressed in bacteria, only the antibody fragments that contain the binding regions are displayed on the surface of the bacteriophage [10]. Most of the phage libraries that have been constructed display the antibody fragment on the surface of the phage minor coat proteins (pIII). It has been shown that both Fab [27,45] and scFv [11,13,23] can be expressed in the surface of M13 without apparent loss of the antibody's specificity and affinity. Since the scFv format has been shown to be more efficient for display on phage coat [33] and the aim of this research was to develop the most efficient method to construct a compact human antibody, the scFv format was chosen.

Several strategies have been described for producing antibody phage display library in scFv format but it is difficult to define the minimal repertoire size needed to retrieve good binders to antigens. Many approaches have been proposed to improve phage displayed human antibody repertoires, mainly by increasing the library sizes [17,35], by sophisticated *in vivo *(Cre-*lox*) recombination method [17], and by improving cloning steps [33,35]. In this study, we describe a simple and highly efficient method to construct a compact human scFv phage library, by modification of several previously published protocols [15,32,46]. Our method needs the least number of oligonucleotide primers (33 primers). The lab of J.D. Marks used 66 primers to construct a naive library [13] while the lab of J. McCafferty used 89 primers [44]. In these reports, two sets of primers were used to construct the library. The first set allow the amplification of the V_H _and V_L _gene repertoires from cDNA, and the second set is used to introduce restriction sites for sub-cloning the genes into phagemid. In our work, we optimized the protocol such that only one set of primers are used to both amplify the genes and introduction of restriction sites, thus reducing the number of primers by half. Moreover, in all of the previous reports on the construction of large (10^9–10^) naïve human libraries, smaller libraries were first created and then combined together to obtain the larger libraries. For example, Vaughan's library of 1.4 × 10^10 ^was generated from three sub-libraries [35], Sheet's library of 6.7 × 10^9 ^was generated from two sub-libraries [33], and McCafferty's library of 10^10 ^was generated from sixteen sub-libraries [44]. In our work, because of the compact size, we used only a couple of ligation reactions and electroporations, compare to thirty six [33] to several hundred electroporations [35], and more than twelve ligation reactions [33] used by others to construct the libraries. Thus, one can use our method to create a compact phage display antibody library that is better than other libraries of the same size, and equally well to larger libraries, with less time and budget.

The scFv phage library was created by focusing on amplification of highly diverse re-arranged V genes, as it has been formerly suggested that the most useful scFv library are constructed from V-genes rearranged *in vivo *[33]. In order to reduce amplification bias we performed 75 independent PCR reactions using all possible combinations within a primer set, modified from previous reports [14]. The high diversity of this compact library came from the high diversity of re-arranged variable region genes, which were isolated from one hundred and forty non-immunized healthy donors. This is the highest number of re-arranged V-gene templates that has ever been reported. Other reports of large and complex human scFv libraries, for example, used five [33], forty two [44], forty three [35], or fifty [47] donors.

The cDNAs from peripheral blood lymphocytes of the 140 non-immunized donors were synthesized using a mix of random hexamers and oligo dTs, so that all five antibody classes could be represented. Amplification using these non-specific primers has been shown to be as efficient as using germline, V_H_, IgG or IgM, specific primers for the construction of efficient naïve repertoires [13,48]. In order to create scFv fragment genes as a V_H_-linker-V_L _type, the 3' ends of V_H _genes were made complementary to 5' ends of V_L _genes through a (Gly_4_Ser)_3 _linker peptide, and the V_H _and V_L _genes were assembled and amplified by overlap extension followed by pull-through PCR. Moreover, to ensure the correct overlaps during assembly PCR, the three (Gly_4_Ser)_3 _repeat in the single chain linker region were encoded by different codons (Figure 2). A proof reading DNA polymerase was used in the overlap extension step to ensure the accurate joining of blunt ends of V_H _and V_L _genes segments and in-frame formation of scFv repertoire. For other steps, TAQ DNA polymerase was used due to a more efficient amplification using this polymerase. In addition, the mutations created by amplification errors could add more diversity to the library. The primer sets used for the amplification of V gene repertoires were designed based on information retrieved from V-BASE [34] and previous publications [14,32]. The 5' and 3' ends of each primer incorporated sequences which could be cleaved by restrictions enzymes, avoiding a two step amplification procedure as previously reported for a murine scFv library [32]. It has been previously suggested that several hundred bps of DNA sequence 5' and 3' of the scFv cloning sites is required for efficient digestion of the insert [32,33]; however we found that this is not necessary, provided that the inserts were incubated with the restriction enzyme for at least 10 hour. In addition to the shortest list of primer used, the fewest steps were needed for the generation of the library. This is because only one ligation step and two electroporation were required, without the creation of sub-libraries as performed by other strategies [33,35,44].

Utilization of different modified helper phages to improve the bio-panning efficiency has been reported [37,49-54]. Here, the helper phage KM13 [37], which is a protease sensitive helper phage, was used during the bio-panning. This modified helper phage encodes a modified gene III, encoding a peptide sequence which will be cleaved by trypsin, between domain D2 and D3. By applying trypsin cleavage following selection of the phage antibody library, the phage particles not carrying an antibody-pIII fusion would lose all there domain D1 and D2 of protein 3, and thus lose their ability to infect bacteria; whereas phage particles carrying an antibody-pIII fusion would retain their infectivity, due to the absence of the protease sensitive site in the gene III of the fusion. Thus, in the bio-panning procedure described here, elution was done by a combination of low pH treatment with glycine buffer (pH2.2) and trypsin treatment. Any phagemid encoded phage display library contain a large proportion of phage particles devoid of pIII fusion on their surface, however these can still be retrieved following selection due to non-specific binding of phage particles. Removal of phage particles carrying no scFv by trypsin digestion, greatly improve the efficiency of the selection. We found that we obtained better result when using KM13 helper phage than regular helper phage, M13K07 (data not shown). Utilization of KM13 helper phage also limits the number of eluted phages after the first round of section [55], which would be a logic consequence of decreasing the number of background binders of non-displaying phage particles and taken together this explain why only one round of panning is sufficient for most selections done in this study.

Our compact scFv antibody phage library of 1.5 × 10^8 ^scFv repertoire rendered binders to seven different antigens. The number of different antibody fragments selected with each antigen was in the same range as from other naïve scFv phage display library, even though those libraries are 93 [14] and 44 times [56] larger than ours. Since we aimed to amplify as many different variable regions as possible, sequence diversity was confirmed in both the primary library and the binders retrieved after antigen selection. The high diversity of our compact scFv library was confirmed by DNA sequence analysis of ten randomly picked clones from unselected library and seven selected clones. Sequence analysis of the primary library indicated that variable regions were derived from all six V_H _and seven V_L _gene families. The variable regions derived from V_H_1, V_H_3, V_H_4, V_κ_1, V_κ_3, V_λ_1, V_λ_6 families had been commonly observed among antibody fragments from phage display libraries [33,57], and human hybridomas [58]. Besides variable regions derived from these preferences gene families, other less frequently used V gene families i.e., V_H_2, V_H_5, V_H_6, V_κ_2, V_κ_4, V_λ_2, V_λ_3 and V_λ_5 were also found in our library. These clones were derived from a number of germ line segments with varied number of amino acid substitutions, ranging from 0–24, indicating excellent diversity in our scFv phage library. We observed a preference usage of certain V gene segments in unselected library (V_H_3DP47, V_λ_DPL16, and V_κ_3DPK22) as well as in the selected clones (V_H_3DP47, and V_κ_1DPK9). This is in accordance with the bias of gene segments most often found in nature [17,33,35] The length (5–19) and amino acid sequence of CDR3 regions varied considerably, even when the genes came from the same germ line segments. None of these clones shared the same CDR3 sequence. These are all characteristic of the creation of naturally occurring immunoglobulin repertoire [17,35], which involve somatic recombination during the development of B cells in the central lymphoid organs, and somatic hypermutation that operates on B cell in peripheral lymphoid organs, resulting in an affinity maturation of the antibody population [59]. Thus, a highly diverse and compact antibody library similar to nature could be successfully constructed from a large number of V gene repertoire templates. It has been estimated that the human antibody repertoire is at least 10^11 ^[59]; however, the number of antibody specificities present at any one time is limited by the total number of B cells in an individual, as well as by each individual's encounters with antigens, which is approximately 10^8 ^different specificities at any one time [59]. This is the same diversity of the compact library described in this paper. Therefore, by performing one-round of bio-panning with this library, one can mimic natural selection and obtain a diverse pattern of binding antibodies.

Even if the size of the library is essential for successful isolation of high affinity antibodies, a very large library is difficult to maintain [10] and in many case, high affinity is not necessary the pre-requisite for successful application of antibody. This is because other properties such as specificity, expression level and stability are also important. The affinity of the antibody can be further enhanced by affinity maturation as previously reported [7,60-65]. These methods involve introduction of diversity into the antibody genes by various methods of mutagenesis such as error-prone PCR, DNA or chain shuffling, or oligonucleotide-directed PCR, while affinity selection of the variants with decreasing amounts of antigens [16]. The affinity maturation process is useful either for improving the affinity of antibody that is selected from naive phage library of medium size (10^7–8 ^different clones) that has lower binding affinity, or for generating "super" antibody to be used in certain applications, such as diagnostic or immunotherapy. It has been reported that phage antibodies with approximately tenfold higher affinities (10^11 ^M^-1^) [63,66,67] than the ceiling affinity that can be obtained from *in vivo *selection of B-cell (10^10 ^M^-1^) [65] have been made by in vitro affinity maturation.

The ability to isolate an antibody that can recognize free Aflatoxin from a simple bio-panning procedure underscores the high quality of our compact library. It has been well known that it is difficult to obtain antibody against free haptens [65], especially from the naïve library. Recombinant scFv against soluble alfatoxin that has been generated so far could only be obtained from either an optimized bio-panning method by elution with soluble haptens [39], or isolated from hybridoma [41]. The ability to isolate antibodies that are specific to both conjugated and free Aflatoxin demonstrated the high quality of this compact library. It is possible that the population of donors have been previously exposed to Aflatoxin as it is a very common contaminant in the region.

In addition to hapten, we also demonstrated successful selections of specific antibodies against complex antigens, i.e., crude snake venom, cancer cell surface, and rabies virus, which are more difficult to obtain. This is because of the limited amount of target antigen present in the mixture, the background binding, and enrichment of phage antibodies specific for non-relevance antigens [10]. However, one cannot rule out the possibility that the apparent specific binding of antibody to complex target is actually resulted from the different level of expression of the recognized antigen. Thus, characterization of these selected antibodies, as well as more investigation to determine the identities of their specific partners, are needed to be done.

## Conclusion

In conclusion, a simple method for the construction of a compact and efficient human scFv phage library has been reported. The key to the success of this method is a very high diversity V-gene repertoire template that was obtained from 140 non-immunized donors in combination with optimized primer set and cloning technique. This method is very practical and should be able to be carried out in any academic institutions, small research facilities, and non-profit organizations world-wide, for the generation of both naïve and immunized human phage display scFv library.

## Methods

### Antigens

Albumin from bovine serum (BSA) was natural purified protein obtained from Fluka, USA. Crude venom of cobra (*Naja kaouthia*) and green pit viper (*Trimeresurus albolabris*) were purchased from the Thai Red Cross Society, Thailand. Aflatoxin B1-BSA conjugated and soluble Aflatoxin B1 (Sigma, Germany) were prepared from *Aspergillus flavus*. Amylase enzyme Type XII-A (Sigma, Germany) was prepared from *Bacillus licheniformis*. Purified Chick Embryo Cell (PCEC) rabies vaccine strain flury LEP (Chiron Behring, India) were inactivated rabies viruses that were cultured in primary chicken fibroblast cell. Cholangiocarcinoma, cell line (KKU-100), which is an egg-proven *Opisthorchis*-associated cholangiocarcinoma derived from porta hepatic cells [68], was a gift from Dr. Banchob Sripa.

### Construction of pMod1 phagemid vector

Phage 3.2 vector (Maxim Biotech Inc, USA) was used as a basis for the construction of pMod1 phagemid vector. The new vector contains *Sfi*I and *Not*I restriction sites and includes a hexahistidine tag and Myc tag. Two oligonucleotides, Mod1up (5' TCG ACC CAT GGC TCG AGG CGG CCG CAC ATC ATC ATC ACC ATC ACG GGG CCG CAG GGC C 3') and Mod1dn (5' CT GCG GCC CCG TGA TGG TGA TGA TGA TGT GCG GCC GCC TCG AGC CAT GGG 3') were annealed and ligated into the phage 3.2 vector (Figure 1) for the generation of pMod1. The insertion was made at *Apa*I/*Sal*I sites using T4 DNA ligase (NEB, USA). The finished pMod1 vector was amplified by transforming *E. coli *DH5αF', and the phagemid DNA was prepared by miniprep extraction kit (Qiagen, Germany). The integrity of the plasmid was confirmed by automated DNA sequence analysis (Macrogen, Korea).

### Construction of human scFv phage library

#### Generation of scFv gene repertoire

The peripheral blood donations from one hundred and forty healthy, non-immunized donors were collected into four pools, according to different blood groups. These blood samples were tested negative and discharged from the Thai Red Cross Society blood donation unit in Nakhon Ratchasima province. Total RNA was prepared from the B lymphocytes and pooled together. Approximately 2 ml of blood samples from each donor were collected into four pools, according to different blood groups (blood group A, B, O and AB). Thus, a total of approximately 280 ml of blood sample was used. B-lymphocytes were isolated from peripheral blood by using Ficoll plaque reagent (Amersham, USA). Briefly, the diluted blood sample (1:1 of blood per PBS) was carefully layered on top of the Ficoll plaque reagent, and then the two-phase solution was centrifuged at 400 × *g *for 30 minutes. B-lymphocytes were collected from the interface between the two phases. The interface contamination such as platelets and plasma proteins were removed by washing with PBS. Total RNA was extracted from B-lymphocytes by TRIzol reagent (Invitrogen, USA). B-lymphocytes were resuspended in 1 ml TRIzol and incubated at 65°C for 15 minutes with occasional inversion of the tube. After adding 0.2 ml of chloroform, the tube was vortexed for 15 seconds and then centrifuged at 12,000 × *g *for 15 minutes at 4°C. The aqueous phase was transferred to a new tube containing 1 μl of RnaseOut (40 U/μl, Invitrogen, USA), and 0.5 ml of isopropanol was added to precipitate RNA. The tube was incubated at room temperature for 10 minutes. The precipitated RNA was pelleted by centrifugation at 12,000 × *g *for 15 minutes at 4°C. The pellet was washed with 0.5 ml of 75% ethanol and then centrifuged at 12,000 × *g *for 15 minutes at 4°C. After the supernatant was removed, the pellet was air dried for 5 minutes at room temperature and dissolved in sterile deionized water. Then 1 μl of RnaseOut (40 U/μl, Invitrogen, USA) was added into total RNA and stored at -70°C. First strand cDNA was generated from 10 μg of total RNA, using MMuLV reverse transcriptase (NEB, USA) with a mix of 20 μM of oligo-dT_18 _and 8 ng of random hexamer primer. The genes for variable regions of heavy chain, κ light chain, and λ light chain (V_H_, V_κ_, and V_λ_) were amplified separately and recombined by three subsequent PCR reactions. The first set of PCR consists of 75 independent reactions to generate variable domains of the heavy and light chains. The heavy chain 5' primers were designed to include a *Sfi*I site, and the light chain 3' primers include a *Not*I site. Light chain 5' primers were designed to include part of the linker region (Gly_4_Ser)_3 _and compatible with the heavy chain 3' primers (Table 1). Each variable region gene was separately amplified using hot start PCR in a reaction of 50 μl containing 5 μl cDNA and 1 μM of each 5' and 3' primer. This reaction was performed using the *Taq *polymerase (NEB, USA). The samples were heat at 94°C for 5 min, followed by 35 cycles of 94°C for 1 min, 55–65°C for 1 min, 72°C for 2 min. The final extension was performed at 72°C for 10 min. Equal amount of PCR products were pooled into collections of V_H_, V_κ_, and V_λ _gene repertoire, and purified from the low melting temperature agarose gel according to standard protocol [69]. In the second PCR, heavy and light chains were assembled and amplified using *pfu *DNA Polymerase (Promaga, USA). The assembly PCR reaction contained equal molar mixture of the pooled heavy (V_H_) DNA and pooled light (V_κ_, or V_λ_) gene repertoire. The assembly reaction was cycled 5 times (94°C for 45 s, 60°C for 50 s, and 72°C for 60 s) without primers. The third reaction created a full-length scFv gene repertoire from the second PCR by PCR amplification in the presence of pull-through primers. This PCR extended the scFv gene from the *Sfi*I and *Not*I sites flanking scFv genes, using the following primers: PTfw (5'-CCT TTC TAT GCG GCC CAG CCG GCC ATG GCC-3') and PTrv (5'-CAG TCA TTC TCG ACT TGC GGC CGC ACG-3'). The reaction was performed using the *Taq *polymerase and 1 μl of assembled products from the second PCR. This pull-through PCR was cycled 30 times (94°C for 1 min, 60°C for 1 min, 72°C for 2 min), and a final extension at 72°C for 10 min. Then the samples were purified by a QIAquick PCR Purification Kit (QIAGEN, Germany) for the next step.

#### Cloning of the scFv into pMod1 vector

The amplified scFv DNA and pMod1 phagemid vector were sequentially digested with *Not*I and *Sfi*I, by incubating at appropriate conditions for 10 hrs. The cut vector was then de-phosphorylated with calf intestinal phosphatase (NEB, USA) and gel-purified using Wizard^® ^DNA Clean-Up System (Promega, USA) before ligation. A total of 2.8 μg of digested scFv DNA were ligated into 5.5 μg of pMod1 phagemid vector, at a vector: insert molar ratio of 1:3, to generate the scFv-gene III fusion library. The ligation was done using T4 DNA ligase (NEB, USA) overnight at 16°C. Ligated DNA populations were electroporated into *Escherichia coli *(*E. coli*) TG1 (Maxim Biotech Inc, USA) using an Eppendrof 2510 electroporator (Eppendrof, USA). The transformed cells were then incubated for 1 hour at 37°C before spreading on TYE plate containing ampicillin (100 μg/mL) and glucose (1% w/v), the plate was incubated overnight at 37°C. Complexity of the library was determined at this step by serially diluting the transformed cells and counting the number of colonies. Ligation efficiency was also determined by counting the number of colonies from no-insert ligation. Colonies were then collected, mixed with glycerol, and stored at -80°C. The library stock was grown to log phase and rescued with M13KO7 helper phage (Maxim Biotech Inc, USA). Recombinant phage preparations were purified and concentrated by polyethylene glycol (PEG) precipitation before keeping at -80°C.

### Determination of library size

A total of 8.3 μg of DNA was electroporated in to *E.coli *TG1 to generate the scFv phage library. After electroporation the cuvette was flushed with 6 ml of SOC medium and transformed cells were incubated for 1 hour at 37°C before spreading on TYE plate containing ampicillin (100 μg/mL) and glucose (1% w/v), then the plate was incubated overnight at 37°C. Complexity of the library was determined by serially diluting the transformed cells and counting the number of colonies. A volume of 100 μl from transformation reactions was taken and a four step 10-fold serial dilution was made. The 100 μl of each dilution was plated out. The appearance of 250 individual colonies from 10^4 ^dilution titer indicated a library diversity of 1.5 × 10^8^.

### Selection of phage antibody library

Selection of phage particles displaying specific scFv fragments were performed on Immuno 96 MicroWell™ Plates (Nunc, Denmark). The different protein antigens (50–600 μg/ml) in phosphate-buffered saline (PBS) (or 0.1 mM NaHCO_3_, in case of amylase) were coated on the plates overnight at 4°C (in case of Rabies, the preparation was first incubated at 37°C for 2 hrs). For cholangiocarcinoma cell surface, approximately 5 × 10^5^cells growing in a 5-ml flask was used. Following blocking with 2% (w/v) skimmed milk powder in PBS (2% MPBS), a library containing between 10^11 ^and 10^12 ^phage particles were added and the plate was incubated for 2 hours at room temperature (RT; 25–28°C). Non-bound phages were eliminated by washing 10–20 times with PBS containing 0.1% Tween 20 (PBS-T), followed by 10–20 times washing with PBS. The bound phages were eluted by incubation with 50 μl of 1 μg/μl trypsin for 10 min, followed by 50 μl of 50 mM glycine- HCl pH 2.0 (immediately neutralized with 50 μl of 200 mM NaHPO_4_, pH7.5 after 10 min). Eluted phages were used to infect exponentially growing *E.coli *TG1 cells by incubating for 30 min at 37°C. Infected cells were spread on TYE plate containing ampicillin (100 μg/mL) and glucose (1% w/v), then the plate was incubated overnight at 37°C. Individual phage-infected colonies were picked and grown for production of phagemid particles in 96-well plate. The culture was rescued using either M13KO7 or KM13 helper phage (MRC HGMP Resource Centre, Cambridge, UK) as describe elsewhere [55]. Rescued phage particles were used to test their antigen recognition properties by ELISA or to initiate subsequent rounds of selection using the similar conditions. Between one and two rounds of selection were performed for each antigen.

### ELISA screening of selected clones

In order to detect antigen recognition, Immuno 96 MicroWell™ Plates were coated with approximately 5–200 μg/ml of each antigen. For cholangiocarcinoma, the cells were fixed with 4% paraformaldehyde in 96-well tissue culture plate. After overnight incubation at 4°C, plates were blocked with 2% MPBS for 1 hour at RT followed by three washes with PBS. The selected phage preparation was diluted 1:2 in 4% MPBS before adding into each well, and incubated for 1 hour at RT. The plates were washed three times with PBS-T, followed by three times with PBS, and incubated with a 1:5,000 dilution of a mouse anti-M13 phage-horseradish peroxidase (HRP) conjugate (Amersham-Pharmacia Biotech, Sweden) in 2% MPBS. The plates were washed again as described earlier. The ABTS (2,2-azino-di-3-ethyl-benzthiazoine-6-sulfonate) peroxidase substrate (Fluka, USA) was added, and the absorbance was read at 405 nm, using a Sunrise absorbance reader (TECAN, Austria).

### Inhibition ELISA

The inhibition ELISA was performed as described in the normal ELISA method, except that the phage particles were pre-incubated in the presence of increasing amount of soluble Aflatoxin B1 from 0.039–5.0 μg/ml.

### DNA fingerprint analysis and DNA sequencing

The diversity of the selected scFv clones was analyzed by comparing restriction enzyme digestion patterns, a procedure called DNA fingerprinting. The scFv sequence of individual clones was amplified by PCR using the following primers: PTfw (5'-CCT TTC TAT GCG GCC CAG CCG GCC ATG GCC-3') and PTrv (5'-CAG TCA TTC TCG ACT TGC GGC CGC ACG-3'). The amplified product was digested with a frequent cutting enzyme, *Bst*NI (NEB, USA) and analyzed on 2% agarose gels. For DNA sequencing, plasmid DNA from different clones was purified using MiniPreps kit (QIAGEN, Germany) and the inserts were sequenced using the dideoxynucleotide chain-termination method with -96gIII primer (5'-CCC TCA TAG TTA GCG TAA CG-3'), corresponding to the vector sequence downstream of the scFv gene. After the amino acid sequences were translated, they were analyzed by using IgBLAST [36] and V BASE [34] software.

### Soluble scFv antibodies production

*E. coli *HB2151 (Maxim Biotech Inc, USA) cells carrying the phagemid encoding the scFv antibody were grown in 10 ml of 2 × TY medium containing ampicillin (100 μg/mL) and glucose (1% w/v). After reaching an OD_600 _of 0.9, cells were harvested and grown at 30°C in glucose free 2 × TY medium containing 100 μg/mL ampicillin and 0.1 mM isopropyl-μ-d-thiogalactopyranoside (IPTG). The cell culture supernatant containing scFv were collected after induction for 20 hours. In some case, the cell lysate containing scFv were extracted after induction for 6 hours.

## Authors' contributions

PP is the PhD student at school of Biotechnology, Suranaree University of Technology, under the supervision of MY, and co-supervised by PK. NJ and KR are graduate students under the supervision of MY. PP constructed the library. PP, NJ and KR participated in affinity selections and characterizations of scFv to different targets. All authors read and approved the final manuscript.

## Supplementary Material

Additional File 1 **Amino acids sequences of ten random clones from unselected library.** Amino acid sequence and germ line family of ten random clones from unselected library. Click here for file
