# Supplementary material for: A compact phage display human scFv library for selection of antibodies to a wide variety of antigens
Source: BMC Biotechnol. 2009 Jan 29;9:6. doi: 10.1186/1472-6750-9-6 (PMC2642811; doi:10.1186/1472-6750-9-6)
Supplement: Additional File 1 — Amino acids sequences of ten random clones from unselected library. Amino acid sequence and germ line family of ten random clones from unselected library. [file 1472-6750-9-6-S1.pdf]

Amino acids Sequences of ten random clones from unselected library

Clone	FR1	CDR1	FR2	CDR2	FR3	CDR3	Germline	Amino acid different from germline	Family
**VH**									
1	QVQLQQSGPGLLKPSQTLSLTCAISGDSVS	SKGPAWN	WIRQSPSRGLEWLG	RTYYWSGWRHDYAPSLQS	RITINPDTSKNQFSLQLNSVTPDDTAVYYCAK	GRDSGFDI	IGHV6-1*01 (DP74)	12	VH3
2	VQLVESGGGLVKPGESLRLSCEASGFA FS	SYNMN	WLRQAPGKGLEWVS	SMSPSGRDIFYPESLKG	RFTASRDNARNSLYLQMNSLRVEDTAMYYCVR	STLLEIAGKSPGLDM	IGHV3-21*01 (DP77)	20	VH3
3	QVNLRESGPTQVKPTQALTLTCTVSGVSLS	TSGVGVG	WLRQAPGKGLEWLA	LLYWDDDTRYNPSLKT	RLAITRGSSRDQVVLTVTNVDPSDTGTYFCAH	RRSGLLVFGIRDAFDI	IGHV2-5*09 (S12-2)	24	VH2
4	VQLQQSGAEVKKPGSSVKVSCKASGGTFS	SYAIS	WVRQAPGQGLEWMG	GIIPIFGTANYAQKFQG	RVTITADESTSTAYMELSSLRSEDTAVYYCAR	VRDTAMEFFDY	IGHV1-69*12 (DP10)	2	VH1
5	QVQLQQSGPGLVKPSETLSLTCIVSGGSIT	SYSWS	WTRQPAGQGLEWIG	RIYNGGSTNYNPSLKS	RVTMSLDTSKNQFSLRLSSVTAADTAVYYCAR	GPYGTGSQCHVFDP	IGHV4-4*07 (VIV4)	2	VH4
6	QVQMQESGAGLLNPLETLSLTCTVYGGSVS	NHYYYGS	RIRQPPGKGQEWFA	YIHYTETTNYNPSLKS	RVTISVDTSKNQFSLRLSSVTAADTAVYYCAR	VDILSGMRHF	IGHV4-61*08 (DP66)	19	VH4
7	EVQLVESGAEVRRPGESLTISCRGSGDTFP	KHWIA	WVRQMPGKGLEWLG	RIDPSDSYTNYNPSFQG	HVSISADQSISTVYLQWSSPKASDTALYYCAR	LTCRTTSCYTDNWSDA	IGHV5-a*01 (VH32)	17	VH5
8	QVQLQQSGPGLVKPSQTLSLTCAISGDSVS	SNSAAWN	WIRQSPSRGLEWLG	RTYYRSKWYNEYALSVKS	RLTINPDTSKNQFSLILNSVTPEDSAVYYCAT	WRFDY	IGHV6-1*01 (DP74)	5	VH6
9	EVQLVESGGGLVQPGGSLRLSCAASGFTFS	SYEMN	WVRQAPGKGLEWVS	YISSSGSTIYYADSVKG	RFTISRDNAKNSLYLQMNSLRAEDTAVYYCAR	VSLDTAGDAFDI	IGHV3-48*03 (DP58)	0	VH3
10	VQLVQSGGGLVQPGGSLRLSCAASGFTFS	SYAMS	WVRQAPGKGLEWVS	AISGSGGSTYYADSVKG	RFTISRDNSKNTLYLQMNSLRAEDTAVYYCAK	DNTEMVRGVMLYYYYYMDV	IGHV3-23*04 (DP47)	1	VH3
**VL**									
1	DIQMTQSPDSLAVSLGERATINC	KSSQSVLFSSNNKHHLA	WYQQKRGLPPKLLIF	WASTRES	GVPDRFSGSGSGTDFTLTISSLQAEDVAVYYC	QQYQSIPHT	IGKV4-1*01 (DPK24)	9	VK4
2	SSELTQDPAVSVALGQTVRITC	QGDSLRTYYAS	WYQQKPGQAPVLVIY	GKNNRPS	GIPDRFSGSSSGNTASLTITGAQAEDEADYYC	NSRDSSDNHVV	IGLV3-19*01 (DPL16)	2	VL3
3	EIVLTQSPGTVSLSPGQRVTLSC	RASQSVRGSFFA	WYQQKPGQAPRLLIH	GASSRAT	GIPDRFDGSGSGTDFTLSISRLETEDFAVYYC	QQYGTSPYT	IGKV3-20*01 (DPK22)	12	VK3
4	QSALTQPASASGSPGQSVTISC	TGTISDIGAHDLVS	WYQQPPGKAPKLIIF	EVSKRAS	GVPDRFSGSKSGNAASLTISGLQADDEADYFC	CSSTNKNNFAVE	IGLV2-8*01 (V1-2)	19	VL2
5	QAVLTQPSSLSASPGSSASLTC	TLRSDFDVRSYRIY	WYQQKPGSPPQYLLR	FKSDSEKHRGS	GVPSRFSGSKDASANAGILLISGLQFDDEADYYC	MIYYNMASE	IGLV5-45*03 (V4-2)	16	VL5
6	EIVLTQSPLYLPVTPGEPASISC	RSSQSLLHSNGYNYLD	WYVQKPGQSPQLLIY	LGSNRAS	GVPDRFSGSGSGTDFTLEISRVEAEDVGVYYC	MQALQTPK	IGKV2D-28*01 (DPK15)	5	VK2
7	QAVLTQPSSLSASPGASASLTC	TLRSGIDVAAYRIY	WYQQKPGSPPQYLLR	YKSDSDKQQGS	GVPSRFSGSKDASANAGILLISGLQSQDEADYYC	AIWHNSAWV	IGLV5-45*03 (V4-2)	6	VL5
8	LTQPPSVSVSAGQTVSITC	SGENLQKIYVS	WYQQKPGQSPILVIY	KDNKRPS	GIPERFSGSNSGNTATLTVSGALAADEAEYYC	QTWDIDTAL	IGLV3-1*01 (DPL23)	23	VL3
9	SSELTQDPAVSVALGQTVTITC	QGESLRNYFAS	WYQQKPGQAPILVMY	DEDIRPS	GIPDRFSGSSSEKTASLTITGVQAEDEATYYC	KCRGSGGDHLEIL	IGLV3-19*01 (DPL16)	19	VL3
10	NFMLTQPHSVSESPGKTVTISC	TRSSGFIDTNYVQ	WYQQRPGSAPTPVIY	ESKQRPS	GVPVRFSGSVDISSNSASLTISGLETEDEADYYC	QSFDSNKLFV	IGLV6-57*01 (V1-22)	13	VL6

* Analysis of germ line family was done by using Ig BLAST [36], and DNA Plot program [34] (shown in parenthesis).
